# The Role of Persulfide Metabolism During Arabidopsis Seed Development Under Light and Dark Conditions

**DOI:** 10.3389/fpls.2018.01381

**Published:** 2018-09-19

**Authors:** Christin Lorenz, Saskia Brandt, Ljudmilla Borisjuk, Hardy Rolletschek, Nicolas Heinzel, Takayuki Tohge, Alisdair R. Fernie, Hans-Peter Braun, Tatjana M. Hildebrandt

**Affiliations:** ^1^Department of Plant Proteomics, Institute of Plant Genetics, Leibniz University Hannover, Hanover, Germany; ^2^Department of Molecular Genetics, Leibniz Institute of Plant Genetics and Crop Plant Research (IPK), Gatersleben, Germany; ^3^Max Planck Institute of Molecular Plant Physiology, Potsdam, Germany

**Keywords:** persulfide signaling, embryogenesis, amino acids, mitochondria, cellularization, cysteine degradation

## Abstract

The sulfur dioxygenase ETHE1 oxidizes persulfides in the mitochondrial matrix and is involved in the degradation of L-cysteine and hydrogen sulfide. ETHE1 has an essential but as yet undefined function in early embryo development of *Arabidopsis thaliana*. In leaves, ETHE1 is strongly induced by extended darkness and participates in the use of amino acids as alternative respiratory substrates during carbohydrate starvation. Thus, we tested the effect of darkness on seed development in an ETHE1 deficient mutant in comparison to the wild type. Since ETHE1 knock-out is embryo lethal, the knock-down line *ethe1-1* with about 1% residual sulfur dioxygenase activity was used for this study. We performed phenotypic analysis, metabolite profiling and comparative proteomics in order to investigate the general effect of extended darkness on seed metabolism and further define the specific function of the mitochondrial sulfur dioxygenase ETHE1 in seeds. Shading of the siliques had no morphological effect on embryogenesis in wild type plants. However, the developmental delay that was already visible in *ethe1-1* seeds under control conditions was further enhanced in the darkness. Dark conditions strongly affected seed quality parameters of both wild type and mutant plants. The effect of ETHE1 knock-down on amino acid profiles was clearly different from that found in leaves indicating that in seeds persulfide oxidation interacts with alanine and glycine rather than branched-chain amino acid metabolism. Sulfur dioxygenase deficiency led to defects in endosperm development possibly due to alterations in the cellularization process. In addition, we provide evidence for a potential role of persulfide metabolism in abscisic acid (ABA) signal transduction in seeds. We conclude that the knock-down of ETHE1 causes metabolic re-arrangements in seeds that differ from those in leaves. Putative mechanisms that cause the aberrant endosperm and embryo development are discussed.

## Introduction

Seed development of plants starts with the unique event of double fertilization. The resulting seed compartments, endosperm and embryo, undergo specific developmental sequences with dramatic changes occurring at the cellular and molecular levels during the transition from tissue differentiation and growth to storage deposition during seed filling. The endosperm is essential for embryo development. It embeds the embryo and is surrounded by the seed coat. In *Arabidopsis thaliana* the endosperm is of transient nature and only the aleurone layer remains until seed maturity (Novack et al., 2010). Its development is characterized by a stepwise differentiation including transition from a syncytial to a cellular phase (Brown et al., 1999, 2003; Berger, 2003; Olsen, 2004). The process of cellularization has a great impact on the accurate embryo development (Hehenberger et al., 2012) and conducts the initiation of embryo growth (Brown et al., 1999). During seed maturation the embryo massively accumulates storage compounds within the cotyledons and becomes a highly specialized storage tissue. At early stages starch and hexoses accumulate transiently, but their amount gradually decreases accompanied by a rapid increase of protein and lipid storage (Baud et al., 2002; Borisjuk et al., 2005). The synthesis of storage compounds requires provision of sufficient amounts of precursor molecules, reductants and energy (Fait et al., 2006; Gallardo et al., 2008). Nutrients such as sugars and amino acids delivered from the mother plant are precursors for storage product biosynthesis in seeds (Melkus et al., 2009). An adaption of energy metabolism including photosynthesis and respiration is essential to enable efficient storage product accumulation. Seed plastids hold special structures and have adapted their metabolism to cope with reduced light levels available for photosynthetic reactions (Borisjuk et al., 2004; Borisjuk et al., 2013). The permeability of gasses into seeds is low, and plastidial activity during the maturation phase contributes to oxygen allocation and reassimilation of CO_2_ (Ruuska et al., 2002; Borisjuk et al., 2005; Rolletschek et al., 2005; Borisjuk and Rolletschek, 2009). Highest photosynthetic activity is observed during storage product synthesis, suggesting a correlation of metabolic processes and photosynthesis (Ruuska et al., 2002; Fait et al., 2006). Recently it was postulated that amino acids are not only precursors for storage proteins within seeds, but might also serve as alternative substrates for mitochondrial metabolism during situations of high energy demand (Galili et al., 2014). In vegetative tissues amino acid catabolism is induced by carbon starvation such as periods of extended darkness (Araujo et al., 2010, 2011; Hildebrandt et al., 2015). First studies denote a potential effect of amino acid degradation also on the energy status of seeds (Weigelt et al., 2008; Angelovici et al., 2009, 2011; Gu et al., 2010; Credali et al., 2013). However, biosynthesis as well as catabolism of amino acids in seeds is still largely unknown. The mitochondrial sulfur dioxygenase ETHE1 (AT1G53580) is part of a sulfur catabolic pathway that catalyzes the oxidation of persulfides derived from cysteine or hydrogen sulfide to thiosulfate and sulfate (Höfler et al., 2016). In Arabidopsis leaves ETHE1 has a key function in amino acid catabolism in situations of carbohydrate starvation such as extended darkness (Krüßel et al., 2014). This function might be mediated by the removal of hydrogen sulfide or persulfides, which both can act as signaling molecules in diverse physiological processes (Romero et al., 2013; Gotor et al., 2015; Höfler et al., 2016). In seeds, ETHE1 knock-out leads to alterations in endosperm formation and finally causes seed abortion (Holdorf et al., 2012). It has been shown that a sulfur dioxygenase activity of 1% present in an ETHE1 knock-down mutant (*ethe1-1*) is sufficient for embryo survival, but development is severely delayed (Krüßel et al., 2014), which underlines the importance of this enzyme for seed metabolism.

This study aimed to investigate the physiological role of mitochondrial persulfide oxidation in seeds. One major aspect was to establish whether the functional context of ETHE1 in leaves, the use of amino acids as alternative respiratory substrates, is also relevant for embryo development. Therefore, our experimental approach included shading of the siliques combined with phenotypic analysis, metabolite profiling and comparative proteomics (Supplementary Figure S1). Our results show a strong effect of extended darkness on amino acid metabolism in seeds and indicate an additional role of ETHE1 in ABA signaling and cell structure formation.

## Materials and Methods

### Plant Growth and Seed Harvesting

*Arabidopsis thaliana* wild type (ecotype Columbia) and *ethe1-1* (*SALK_021573*, Nottingham Arabidopsis Stock Centre, University of Nottingham) plants were grown in a climate chamber under long day conditions (16 h light/8 h dark, 22°C, 85 μmol s^-1^ m^-2^ light intensity and 65% humidity). Flowers were labeled at the day of pollination. Subsequently siliques were harvested from 1 to 9 DAP. For dark treatment, siliques were shaded with aluminum foil 24 h after flower tagging while the rest of the plant and control siliques were grown under normal light conditions. Seeds from 8 batches including 10 plants each were pooled and used for shotgun proteomics as well as metabolite analysis via GC/MS.

### Phenotypic Analysis of Seed Samples

Whole-mount preparations were used for microscopic analysis of *A. thaliana* wild type and *ethe1-1* embryo development grown under light and dark conditions. Seeds from at least 5 siliques each were cleared by incubation with Hoyer’s solution [15 mL distilled water, 3.75 g gum Arabic, 2.5 mL glycerine, 50 g chloral hydrate] overnight. Embryo development was analyzed by using Normarski optics and light microscopy. Observed embryo stages were counted. For analyzing seed tissue differentiation histological sections were prepared from 7 DAP *ethe1-1* and wild type seeds grown under light and dark conditions. Seed samples were fixed with 1.25% formaldehyde and 0.1 M phosphate buffer overnight followed by dehydration of the samples by a series of ethanol aqueous solutions (30–99%). After dehydration samples were infiltrated with Historesin (Leica). Sections of 3.5 μm were prepared using a microtome (HYRAX M55, Zeiss) and Sec55 low profile blades (MICROM). Sections were stained with 0.5% Toluidine Blue O in 200 mM phosphate buffer. Photographs of cleared seeds and sections were taken with a Zeiss microscope (Axioskop2) and AxioCam MRc5 camera. Seed size and weight were measured for mature seeds of *ethe1-1* and wild type grown under light and dark conditions for 100 seeds each. Significant differences were calculated using a Student’s *t*-test (*p*-value ≤ 0.05). For electron microscopy of mitochondria seeds of wild type and *ethe1-1* (5 DAP) were fixed and embedded in accordance to Tschiersch et al. (2011). Digital records of seed mitochondria were made on a Zeiss 902 electron microscope at 80 kV.

### Germination Rates

Arabidopsis seeds of wild type and *ethe1-1* grown under light and dark conditions were surface sterilized with 6% sodium hypochloride and 100% ethanol followed by five washing steps with sterilized water. For vernalization seeds were incubated for 2 days at 4°C in the dark. Approximately 20 seeds were sown per plate (3 replicates per sample) on MS-medium [60 mM sucrose, 1% Agar, 0.5% MS-medium (Duchefa), pH 5.7-5.8 with KOH] and MS medium without a carbon source and incubated for another 2 days at 4°C in the dark. Afterwards the plates were placed to a growth chamber (24°C, 16 h light/8 h dark). After 72 h germinated seeds were counted. A seed is considered to be germinated when the radicle ruptures the endosperm and the testa.

### Metabolite Profiling

Metabolites of *ethe1-1* and wild type seeds (4, 5, and 9 DAP) grown under light and dark conditions were extracted and subsequently analyzed by GC-TOF MS as described by Lisec et al. (2006). Chromatograms and mass spectra were evaluated by using TagFinder 4.0 software (Luedemann et al., 2008) and Xcalibur 2.1 software (Thermo Fisher Scientific, Waltham, MA, United States). Metabolites were identified in comparison to database entries of authentic standards (Kopka et al., 2005; Schauer et al., 2005). Peak areas of the mass (m/z) fragments were normalized to the internal standard (ribitol) and fresh weight of the seed samples. Identification and annotation of detected peaks followed recent recommendations for reporting metabolite data (Fernie et al., 2011). Storage products (starch, total proteins, total lipids) and free amino acids of mature wild type and *ethe1-1* seeds grown under light and dark conditions were measured as described in Schwender et al. (2015). Shortly, plant material was freeze-dried and weighed for determination of dry weight. Upon subsequent pulverization, the material was used for (1) the quantification of total lipid content measured using time domain nuclear magnetic resonance (as in Fuchs et al., 2013), (2) analysis of total protein content [measured as total nitrogen^∗^5.64 using elemental analysis (as in Borisjuk et al., 2013)], (3) analysis of metabolites and starch. For metabolites, pulverized material was extracted three-times with ethanol (100%). The ethanol fraction was dried down and was subject to chromatographic separation followed by mass spectrometric detection of sugars and free amino acids (as detailed in Schwender et al., 2015). Starch was determined spectrophotometrically in the pellet remaining after ethanolic extraction (Heim et al., 1993).

### Sample Preparation for Mass Spectrometry

Proteins of wild type and *ethe1-1* (9 DAP, light, dark) were extracted from 30 mg of pulverized seeds (pooled sample) by adding 150 μL extraction buffer [4% SDS (w/v), 125 mM TRIS, 20% glycerol (v/v)] and incubating at 60°C for 5 min. Another 150 μL of ddH_2_O were added to the sample followed by centrifugation at 18000 × *g* for 10 min. The protein concentration of the supernatant was measured by using the Pierce^TM^ BCA Protein Assay Kit (Thermo Fisher Scientific, Dreieich, Germany). To each sample (total protein 50 μg) 2-mercapto ethanol with a trace of bromophenol blue was added to a final concentration of 5%. SDS gel electrophoresis using a 1D glycine gel (stacking gel 4% acrylamide, separating gel 14% acrylamide) was performed according to Laemmli (1970). The gel run was stopped before proteins entered the separation gel. The gel was fixed with 10% (v/v) acetate in 40% (v/v) methanol for 45 min and stained with Coomassie blue CBB G-250 (Merck, Darmstadt, Germany) for 30 min as described by Neuhoff et al. (1985, 1990). Gel bands were cut using a scalpel and diced into 1.0–1.5 mm cubes. Carbamidomethylated followed by tryptic digestion and extraction of proteins was performed according to Klodmann et al. (2010). Resulting peptides were resolved in 20 μL of 2% [v/v] ACN, 0.1% [v/v] formic acid (FA) prior to MS analysis.

### Shot Gun Mass Spectrometry and Relative Protein Quantification

Shot gun mass spectrometry was performed by using a Q-Exactive (Thermo Fisher Scientific, Dreieich, Germany) mass spectrometer coupled to an Ultimate 3000 (Thermo Fisher Scientific, Dreieich, Germany) UPLC. Three times (technical replicates) four microliter of peptide solution per seed sample were injected into a 2 cm, C18, 5 μm, 100 Å reverse phase trapping column (Acclaim PepMap100, Thermo Fisher Scientific, Dreieich, Germany). Peptide separation was done on a 50 cm, C18, 3 μm, 100 Å reverse phase analytical column (Acclaim PepMap100, Thermo Fisher Scientific, Dreieich, Germany). Peptides were eluted by using a non-linear 2% [v/v] to 34% [v/v] acetonitrile gradient in 0.1% [v/v] formic acid of 60 min. For MS analysis a spray voltage of 2.2 kV, capillary temperature to 275°C and S-lens RF level to 50% was tuned. For full MS scans, the number of microscans was adjusted to 1, resolution to 70,000, AGC target to 1e6, maximum injection time to 400 ms, number of scan ranges to 1 and scan range to 400 to 1600 m/z. For dd-MS2, the number of microscans was adjusted to 1, resolution to 17,500, AGC target to 1e5, maximum injection time to 120 ms, Loop count to 10, MSX count to 1, isolation window to 3.0 m/z, fixed first mass to 100.0 m/z and NCE to 27.0. Data dependent (dd) settings were adjusted to underfill ratio of 0.5%, intensity threshold to 2.0e3; apex trigger to 10–60 s, charge exclusion to unassigned 1, 5, 5–8, >8, peptide match to preferred, exclude isotopes to on and dynamic exclusion to 45.0 s. MS/MS spectra were queried against an in-house Arabidopsis protein database also containing frequent contaminations and RNA-editing sites using the Andromeda search engine as part of the MaxQuant (Cox and Mann, 2008) software package. Frequent contaminants (BSA, keratin, trypsin) were removed from the final output lists. MaxQuant analysis was performed using version 1.5.2.8. with the same parameter settings as described previously (Fromm et al., 2016). Label free quantification (LFQ) intensities from the corresponding MaxQuant file were uploaded into the Perseus software (Tyanova et al., 2016) and quantitation matrix was built based on log2-transformed LFQ intensity values. Only proteins with a *P*-value < 0.05 were considered statistically significant.

### *In vitro* Cultures With ABA

*In vitro* cultures were used for germination and growth experiments with 1 μM abscisic acid (ABA). The seeds of the wild type (WT) and mutant (*ethe1-1*) were sterilized and planted together on one plate. As control, the plates contained ½ Murashige and Skoog medium and for the treatment with ABA, this medium was supplemented with 1 μM ABA. The plates were transferred to long day conditions after 2 days of stratification at 4°C in darkness. From 2 to 9 days, the germinated (radicula minimal 1 mm) seeds as well as the seeds, which built at least the first two leaves were counted daily.

## Results

A combination of phenotypic analysis, metabolite profiling and comparative proteomics was used to investigate the function of the mitochondrial sulfur dioxygenase ETHE1 in seeds. Since knock-out of ETHE1 is embryo lethal (Holdorf et al., 2012), the knock-down line *ethe1-1* that was described by Krüßel et al. (2014) was used for all experiments.

### ETHE1 Knock-Down Causes a Delay in Embryogenesis Which Is Further Enhanced in the Dark

**FIGURE 1:**
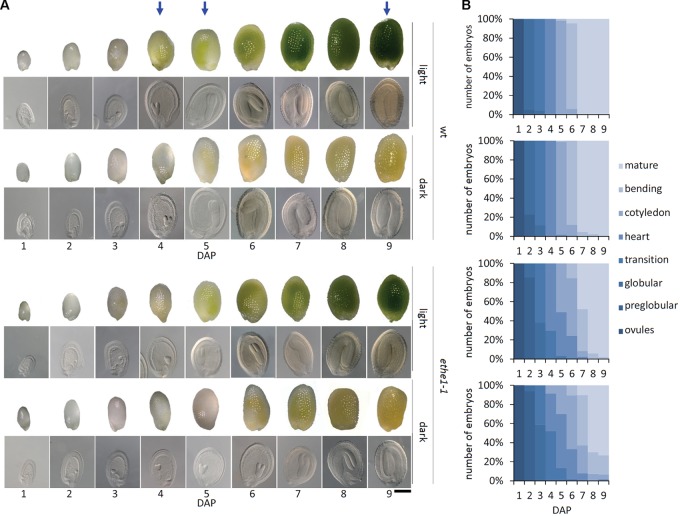
Seed development of *Arabidopsis thaliana* wild type and *ethe1-1* seeds under light and dark conditions. **(A)** Representative seeds and embryos of wild type (wt) and *ethe1-1* from 1 to 9 days after pollination (DAP) grown under light and dark conditions, bars = 100 μm. **(B)** Progression of embryo development in wt and *ethe1-1* seeds. A total of 180 siliques and 7781 seeds between 1 and 9 DAP were analyzed of wild type and *ethe1-1* grown under light and dark conditions. Blue arrows indicates stages investigated by metabolomics and proteomics.

In leaves ETHE1 is strongly induced by extended darkness and has a key function in the use of amino acids as alternative respiratory substrates during carbohydrate starvation (Krüßel et al., 2014). Thus, we tested the effect of darkness on seed development in the *ethe1-1* mutant by covering the young siliques with aluminum foil 1 day after pollination (DAP, Supplementary Figure S1). The role of photosynthesis in seeds is still largely unknown. Since carbohydrates are mainly provided by the mother plant, additional functions compared to leaves can be expected. Therefore, we first tested the effect of darkness on seed development in wild type plants. Overall development of the siliques and the number of seeds produced was not influenced by the shading procedure (data not shown). However, the seeds did not turn green at 4–5 DAP like under light conditions but appeared yellowish (**Figure 1A**). Seeds grown under light and dark conditions were harvested over a period of 1–9 DAP. For each time point (1–9 DAP) and light condition (light, dark) embryo development in seeds from five siliques was analyzed using a seed clearing method. Interestingly, there were no morphological differences between wild type embryos growing under light or dark conditions. Development from globular to mature stage proceeded very uniformly, and all seeds in a silique were at the same developmental stage (**Figure 1B**). In contrast, we observed a delay of embryogenesis in *ethe1-1* seeds with several embryo stages present within one silique. Dark conditions induced a stronger phenotype in *ethe1-1* seeds, which became apparent starting from 3 DAP where most of the embryos were still in the globular stage, whereas light grown *ethe1-1* embryos were in the transition stage. At 9 DAP most of the embryos were fully matured. The developmental delay of *ethe1-1* seeds compared to the wild type observed under control conditions was even more pronounced in darkness.

### Embryogenesis Under Dark Conditions Leads to Reduced Seed Weight and Altered Biomass Composition

**FIGURE 2:**
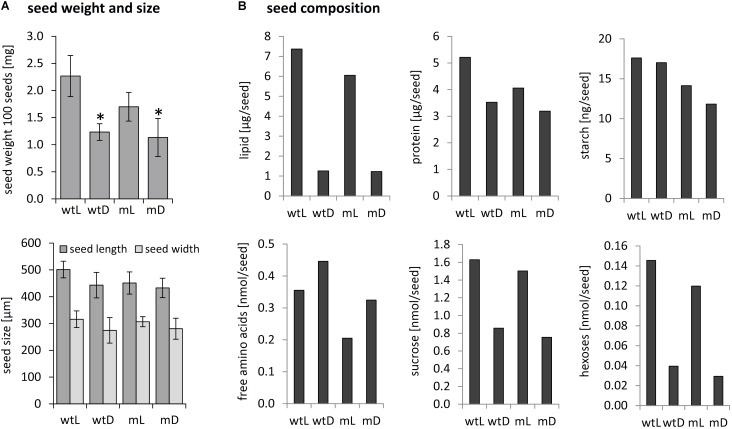
Seed weight, seed size and biomass composition of mature seeds. **(A)** Seed size and weight of 100 mature seeds from wild type and *ethe1-1* plants grown under light and dark conditions. Asterisks indicate significant changes compared to wtL based on a Student’s *t*-test (*p*-value ≤ 0.05). **(B)** Storage compounds and amino acids were extracted from mature seeds from wild type and *ethe1-1* grown under light and dark conditions and quantified. wtL, wild type seeds grown under light conditions; wtD, wild type seeds grown under dark conditions; mL, *ethe1-1* seeds grown under light conditions; mD, *ethe1-1* seeds grown under dark conditions.

Mature seeds of wild type as well as *ethe1-1* plants grown under light or dark conditions were of the same size and morphologically indistinguishable. However, the final seed weight was drastically reduced in darkness compared to control conditions for both genotypes (**Figure 2A**). To investigate whether the lower seed weight was caused by a shift in storage product accumulation, we analyzed the final composition of the mature seeds (**Figure 2B**). For both, wild type and *ethe1-1* seeds, total lipid contents were decreased to about 20% of those found in light grown seeds. Shading of the siliques induced an accumulation of free amino acids, while the protein content was slightly reduced. Concentrations of hexoses and sucrose were strongly decreased after the dark treatment in both wild type and *ethe1-1* seeds, whereas levels of starch remained unaffected. In agreement with the postulated impact of photosynthesis on germination timing (Allorent et al., 2015), we observed a reduced germination capacity of dark grown seeds (Supplementary Figure S2). However, only minor differences were documented on comparison of the biomass composition and germination rate of wild type and *ethe1-1* seeds.

### Darkness as Well as ETHE1 Deficiency Interferes With Seed Amino Acid Homeostasis

Next, we performed comparative metabolite profiling of wild type and *ethe1-1* seeds grown under light and dark conditions. Samples were taken at two different time points plus one additional wild type control (**Figure 1A**, indicated by blue arrows). We selected 4/5 DAP, since the developmental delay in the mutant seeds was most pronounced at this stage. Also, the endosperm still has an important function and covers a large part of the seed. A second set of samples was collected at 9 DAP, when the seed was completely filled by the embryo and there was no morphological difference between the genotypes or light conditions. The complete datasets are available in Supplementary Tables S1, S2.

**FIGURE 3:**
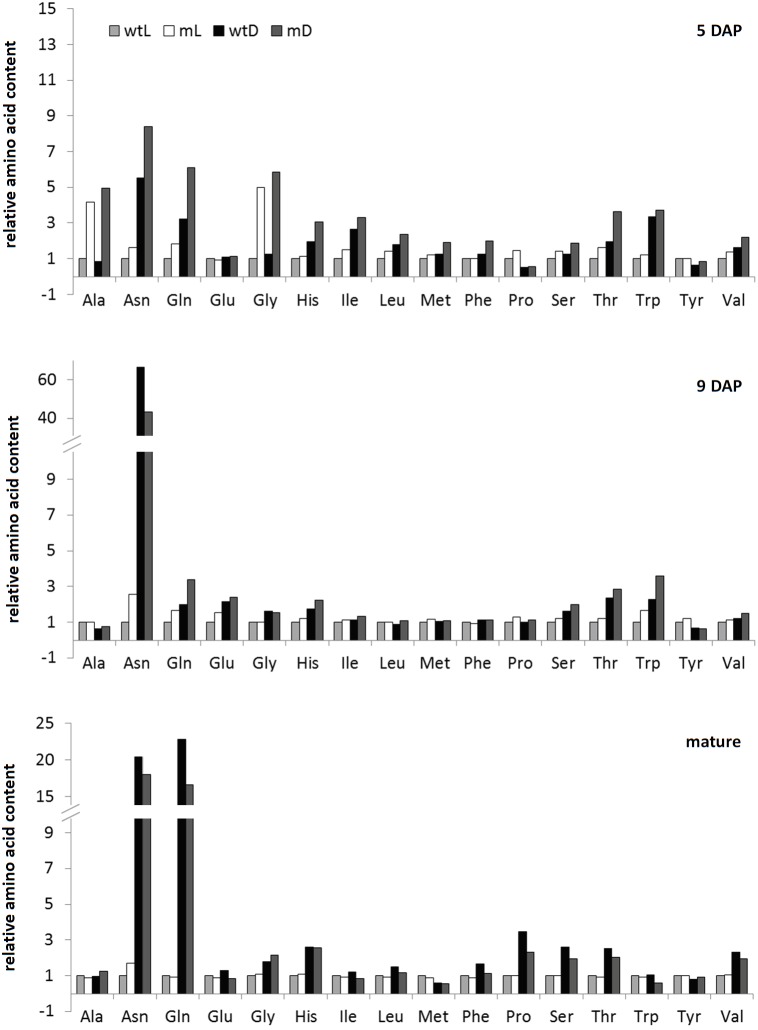
Amino acid profiles. Relative amino acid contents of wild type and *ethe1-1* seeds grown under light and dark conditions at 5 and 9 days after pollination (DAP) and in a mature state. Datasets were normalized to wtL samples of the same age, respectively. wtL, wild type seeds grown under light conditions; wtD, wild type seeds grown under dark conditions; mL, *ethe1-1* seeds grown under light conditions; mD, *ethe1-1* seeds grown under dark conditions.

The strongest metabolic effect of darkness as well as ETHE1 deficiency detectable in the different seed samples was on the level of amino acid profiles (**Figure 3** and Supplementary Tables S1, S2). Contents of the nitrogen-rich amino acids Asn and Gln increased drastically, and they accumulated more than 20-fold in mature seeds grown in darkness compared to control conditions. We also detected a consistent but less pronounced increase in the Thr and His content (about twofold) and transient accumulation of other amino acids such as Trp and the branched-chain amino acids at individual developmental stages. The metabolite profiles of wild type as well as *ethe1-1* seeds revealed only a mild shortage of carbohydrates during development in the absence of photosynthesis (Supplementary Table S1). The level of sucrose, which is the main sugar present at 9 DAP, was nearly identical, and also glucose and fructose, which are dominant earlier in development were only moderately reduced to 65–95% of the level present in seeds grown under light conditions.

While the composition of mature seeds was very similar in the ETHE1 knock-down mutant and in the wild type, specific differences in the metabolite profile became apparent at earlier developmental stages (**Figure 3** and Supplementary Table S1). At 5 DAP Ala and Gly concentrations were increased four–fivefold compared to the wild type under light as well as dark growth conditions. Comparison to the metabolite profile of wild type seeds harvested at 4 DAP confirmed that this effect was not due to the earlier developmental stage present in the mutant. There was a trend toward a general increase in free amino acids in *ethe1-1* seeds compared to the wild type (on average 1.7-fold in the light and 1.9-fold in the darkness) (**Figure 3**). However, the strong accumulation of branched-chain amino acids reported from *ethe1-1* leaves was not present in the seeds. Hexose levels in the mutant were also elevated at 5 DAP, which could, however, be linked to the earlier developmental stage of the *ethe1-1* embryo, since in the wild type hexose concentrations decreased between 4 and 5 DAP (Supplementary Table S1). At 9 DAP the metabolite profiles of the ETHE1 deficient seeds were more similar to the wild type than at the earlier stage. Amino acid concentrations were only slightly increased (on average 1.3-fold), hexose levels were 1.5–2.5-fold higher, and sucrose was decreased to 70% of wild type level in the light and to 60% in shaded siliques. Interestingly, the modified amino acid hydroxyproline, which is part of cell wall glycoproteins, was consistently increased in mutant compared to wild type seeds at 5 and 9 DAP (Supplementary Table S1).

### Effects of Darkness and ETHE1 Knock-Down on the Proteome of Developing Arabidopsis Seeds

**FIGURE 4:**
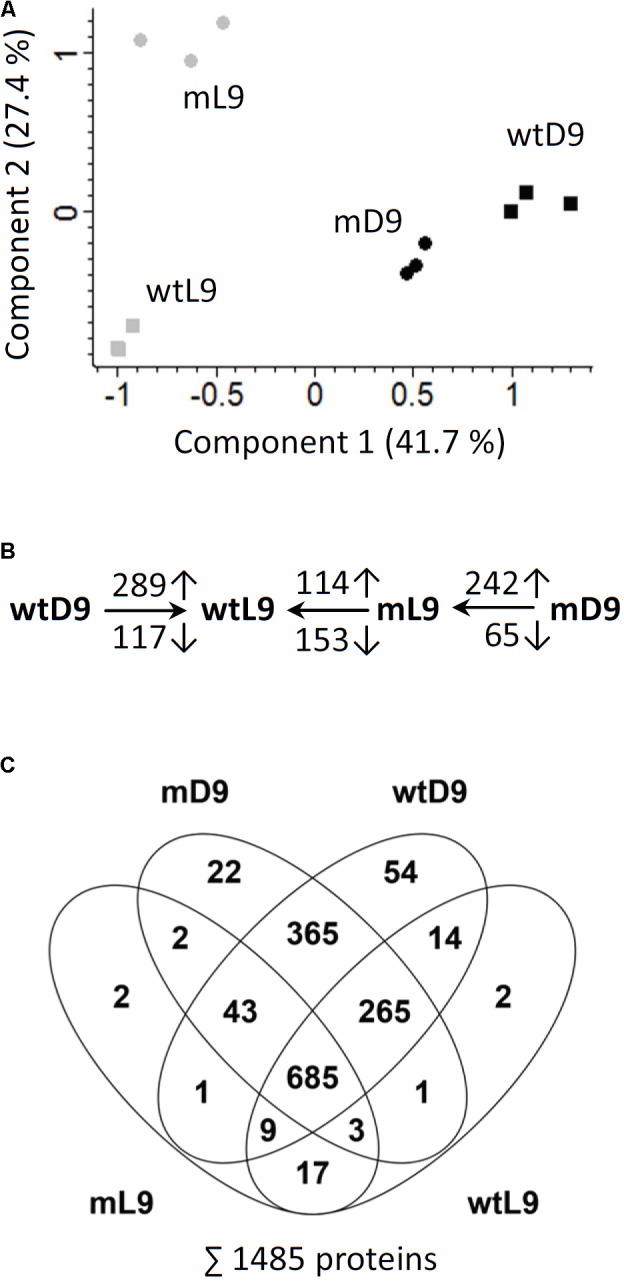
Principle component analysis and overview of proteomic comparisons. **(A)** For quality control of the shotgun MS datasets principle component analysis (PCA) was performed using Perseus software. **(B)** The numbers of significantly increased and decreased proteins for relevant comparisons are indicated (Student’s *t*-test, *p*-value < 0.05). Comparisons were wtD/wtL, mL/wtL, mD/mL. **(C)** Venn diagram illustrating the numbers of proteins detected in the individual sample goups. The total number of detected proteins is listed in the bottom. wtL, wild type seeds grown under light conditions; wtD, wild type seeds grown under dark conditions; mL, *ethe1-1* seeds grown under light conditions; mD, *ethe1-1* seeds grown under dark conditions, numbers indicate the age of the seed in days after pollination.

**FIGURE 5:**
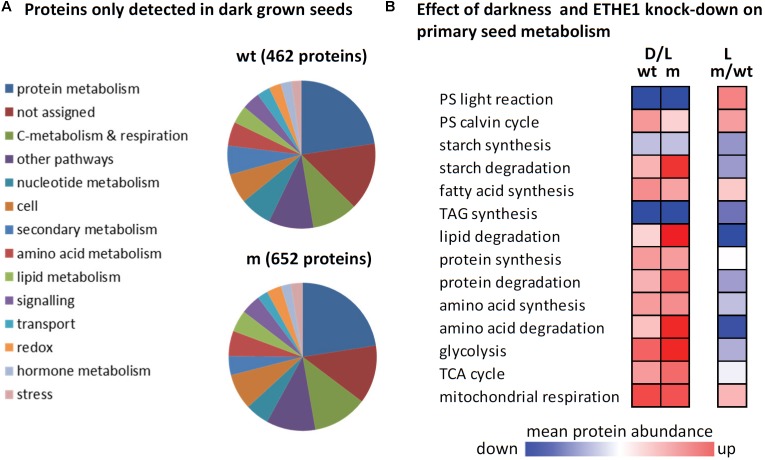
The effect of darkness and ETHE1 knock-down on the proteome of developing Arabidopsis seeds at 9 DAP. **(A)** Functional categorization of proteins that were detected in dark but not in light grown seeds according to the MapMan annotation file (Thimm et al., 2004, version Ath_AGI_LOCUS_TAIR10_Aug2012). **(B)** Log_2_-fold changes in protein abundances were calculated for three different comparisons (from left to right): dark vs. light grown wild type seeds, dark vs. light grown *ethe1-1* seeds, and *ethe1-1* vs. wild type seeds grown under control conditions. The color gradient represents the means of all log_2_-ratios (scaling: –1 to +1) for proteins included in the functional categories listed according to the MapMan annotation file (version Ath_AGI_LOCUS_TAIR10_Aug2012). Wt, wild type; m, mutant; PS, photosynthesis; TAG, triacylglyceride; TCA, tricarboxylic acid.

A shotgun proteomics approach was used to compare protein abundances in wild type and *ethe1-1* seeds grown under control and dark conditions at 9 DAP. In total 1485 unique proteins were identified and the replicates of the samples clustered very well in a principal component analysis (**Figure 4A** and Supplementary Table S3). The proteome comparison indicated a large impact of the availability of light on the seed proteome (**Figure 4C**). Many proteins belonging to various metabolic pathways were only detectable in dark grown samples, and most metabolic pathways were up-regulated in the dark including carbohydrate catabolism, protein metabolism, and amino acid metabolism (**Figure 5**). In general, this response was stronger in *ethe1-1* samples than in the wild type. As expected, darkness led to a clear decrease in proteins related to the light reaction of photosynthesis. Interestingly, there was also a strong reduction of enzymes catalyzing the synthesis of triacylglycerides, which is in line with the severely decreased lipid content of dark-grown seeds (**Figure 2B**). In addition, starch synthesis was down-regulated after shading of the siliques whereas its degradation was induced. The comparison between *ethe1-1* and wild type seeds grown under control conditions identified 114 proteins with significantly increased and 153 proteins with reduced abundance in the mutant (**Figure 4B** and Supplementary Table S3). Photosynthesis was clearly induced in *ethe1-1* compared to the wild type, whereas lipid and amino acid catabolism were most stronly decreased (**Figure 5B**).

### ETHE1 Knock-Down Leads to Impaired Endosperm Development

**FIGURE 6:**
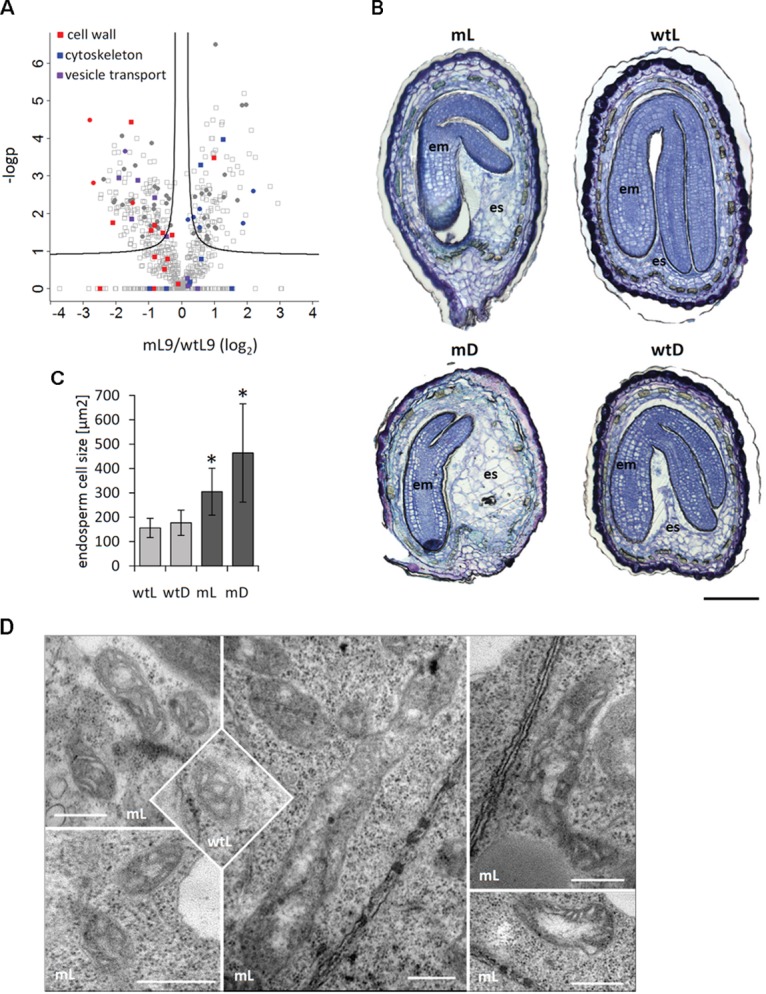
ETHE1 deficiency affects endosperm cellularization. **(A)** Vulcano plot illustrating differences in protein abundance between *ethe1-1* and wt seeds at 9 DAP. Solid lines represent the threshold for significance (FDR: 0.05, s0: 0.1). Proteins involved in cell wall synthesis or degradation are highlighted in red, constituents of the cytoskeleton are highlighted in blue, and proteins involved in vesicle traffic are highlighted in purple. Filled circles represent proteins that are consistenly regulated in light and dark grown *ethe1-1* compared to wt seeds. **(B)** Seed sections (7 DAP) of wild type and *ethe1-1* seeds grown under light and dark conditions. Sections were stained with Toluidine Blue O to visualize endospserm cellularization, bars = 100 μm. **(C)** Endosperm cell size of ca. 50 cells is given as area [μm^2^] obtained from microscopy images using AxioVision software (Version 4.8.1). Asterisks indicate significant changes to wild type light (wtL) based on a Student’s *t*-test (*p*-value ≤ 0.05). **(D)** Electron microsopic analysis of mitochondrial structure from *ethe1-1* seeds compared to wild type, bars = 500 nm; wtL, wild type seeds grown under light conditions; wtD, wild type seeds grown under dark conditions; mL, *ethe1-1* seeds grown under light conditions; mD, *ethe1-1* seeds grown under dark conditions.

A closer look at consistently regulated proteins revealed specific changes in the abundance of proteins related to cell structure (**Figure 6A**). Several isoforms of actin and tubulin were increased in *ethe1-1* seeds compared to the wild type, whereas proteins involved in the synthesis, modulation and degradation of the cell wall as well as constituents of the vesicle transport machinery were reduced. Therefore, we analyzed the cellular structure of developing wild type and *ethe1-1* seeds using Toluidine Blue stained microscopic sections (**Figure 6B**). We observed clear differences in the size of central endosperm cells and calculated the area of about 50 cells per sample type. The average cell size in wild type endosperm was 156 ± 40 μm^2^. In contrast, *ethe1-1* seeds showed characteristically enlarged endosperm cells with a size of 305 ± 96 μm^2^. Darkness further increased the endosperm cell size in the mutant, while endosperm of wild type seeds was not affected (**Figure 6C**).

### ETHE1 Knock-Down Affects Mitochondrial Structure

We next investigated the ultracellular structure of embryo and endosperm cells in *ethe1-1* by using transmission electron microscopy (TEM). The overall organellar structure was relatively normal compared to wild type, with the exception of the appearance of mitochondria (**Figure 6D**). The main population of mitochondria in both wild type and *ethe1-1* was similar in structure (e.g., well distinguished double membrane, lamellar cristae, and relative small area of electron-transparent matrix). The maximal differences in size of mitochondria in embryo of wild type cells (0.08–0.28 μm^2^) and of *ethe1-1* (0.04–0.38 μm^2^) displayed high heterogeneity of population in both genotypes. Similar results were achieved by comparison of mitochondria in endosperm, namely mitochondria size in wild type (0.05–0.42 μm^2^) and *ethe1-1* (0.04–0.34 μm^2^). Extremely high variability of individual organelles did not allow defining any statistically significant differences. However, embryo cells of *ethe1-1* showed aberrant mitochondria, which differed from wild type at the ultrastructural level in means of strongly enlarged size (up to fivefold in length), less pronounced lamellar cristae, larger electron transparent areas in the matrix and some electron-dense insertions.

### ETHE1 Knock-Down Inhibits ABA Signal Transduction

**FIGURE 7:**
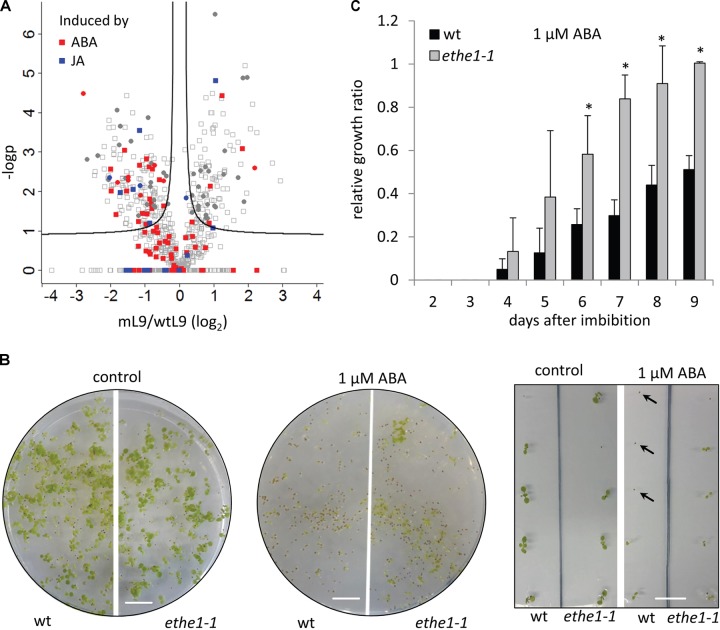
ETHE1 deficiency affects abscisic acid (ABA) signal transduction. **(A)** Vulcano plot illustrating differences in protein abundance between light grown *ethe1-1* and wt seeds at 9 DAP. Solid lines represent the threshold for significance (FDR: 0.05, s0: 0.1). Proteins induced by ABA or jasmonic acid (JA) are marked in red and blue, respectively (Goda et al., 2008). Filled circles represent proteins that are consistenly regulated in light and dark grown *ethe1-1* compared to wt seeds. **(B)** Phenotype of wild type and *ethe1-1* seedlings grown on agar plates containing 0 μM (control) or 1 μM ABA 10 days after transfer to light conditions. Arrows indicate seeds that had germinated but arrested growth after emergence of the radicle. scale bars = 1 cm. **(C)** Relative growth ratio of wt compared to *ethe1-1* seedlings on agar plates containing 1 μM ABA calculated as the percentage of germinated plants that had also developed leaves. All data points were normalized to the growth ratio of *ethe1-1* seedlings at 9 days after imbibition. Asterisks indicate significant differences to the wild type based on a Student’s *t*-test (*p*-value ≤ 0.05).

Defects in endosperm cellularization have also been described for mutants with altered levels of the plant hormone ABA (Cheng et al., 2014; Sreenivasulu et al., 2010). Since ETHE1 expression is strongly induced in ABA treated Arabidopsis seedlings (Supplementary Figure S3, eFP Browser, Winter et al., 2007), we decided to investigate, whether ABA signaling might be affected in *ethe1-1* seeds. ABA inducible proteins were mostly down-regulated in *ethe1-1* compared to wild type seeds at 9 DAP (**Figure 7A**), which might be explained by either lower levels of the signaling molecule or a defect in ABA signal transduction. ABA insensitive mutants can be identified by their ability to grow on ABA containing agar plates, since in the wild type ABA delays seed germination and inhibits early seedling development (Chen et al., 2008). Germination was equally delayed in the presence of 1 μM ABA in wild type and *ethe1-1* seeds, but after 8 days the number of germinated seeds was comparable to the control without ABA for both genotypes. However, while most of the wild type plants arrested growth immediately after emergence of the radicle, several *ethe1-1* seedlings continued growing and developed leaves (**Figure 7B**). The relative growth rate in the presence of exogenous ABA was significantly (about twofold) higher in *ethe1-1* seedlings than in the wild type (**Figure 7C**). In contrast, leaf development was slightly delayed in *ethe1-1* compared to wild type seedlings on the control plates without ABA (Supplementary Figure S4). Almost all of the wild type seedlings had developed leaves already 3 days after imbibition whereas *ethe1-1* plants took 6 days to reach this stage.

## Discussion

The sulfur dioxygenase ETHE1, which is involved in sulfide and cysteine catabolism, has an essential but as yet undefined function in Arabidopsis seed development. In this study, metabolomics and shotgun mass spectrometry based proteomics were implemented to (i) investigate the specific impact of changing light conditions on seed metabolism and (ii) identify the role of persulfide metabolism during seed development.

### Impact of Changing Light Conditions on Seed Metabolism

#### Darkness Reduces the Seed Filling Capacity

In several dicotyledonous species such as Arabidopsis embryos become green during development. Seed plastids differ in their structure from those present in leaves, and since carbohydrates are mainly supplied by the mother plant, one can assume that plastidial metabolism is changed as well (Ruuska et al., 2002). Our results indicate that shading of siliques, which obviously prevents photosynthesis, has a great impact on seed storage metabolism. The content of lipid, which normally accounts for 30–40% of the seed dry weight in the oilseed plant Arabidopsis, was strongly reduced in dark grown seeds, while storage proteins were only mildly affected. Flux analysis suggests that seed photosynthesis contributes quantitatively to the production of NADPH and ATP for fatty acid synthesis in *Brassica napus* (Goffman et al., 2005), which is perfectly in line with our results obtained in Arabidopsis. Starch transiently accumulates during early maturation of the embryo with a peak at 10 DAP and might serve as a carbon source for early storage compound synthesis (Baud et al., 2008). Our proteomics dataset indicates that darkness leads to a down-regulation of starch synthesis and a strong induction of starch degradation at 9 DAP, which might also contribute to the drop in storage lipid production. Since we observed a general increase in carbohydrate catabolism and reduced sugar contents, a possible explanation would be that most of the sucrose supplied by the mother plant is devoted toward ATP production to compensate for the lack of photosynthesis. It is very likely that the reduced storage capacity in darkness leads to the lower seed weight we observed, since under light conditions storage product accumulation coincides with increasing seed weight (Baud and Lepiniec, 2009). Similar results were obtained in a previous study on *B. napus* seeds grown in darkness (Borisjuk et al., 2013). An alternative approach to study the role of photosynthesis in seed development using DCMU to inhibit photosystem II showed no effect on storage protein or lipid content (Allorent et al., 2015). Therefore, it can be assumed that darkness applied to the growing seeds might cause additional effects on development and metabolism.

#### Darkness Induces an Accumulation of Nitrogen-Rich Amino Acids but no Carbohydrate Starvation Response

Nitrogen loading of the seeds is achieved via the import of amino acids (Asn, Gln, and Ala), which during seed filling are mainly used for storage protein synthesis (Higashi et al., 2006). We observed that the nitrogen-rich amino acids Asn and Gln were most dramatically increased under dark conditions. One reason for this effect could be an induction of their synthesis in the absence of light. In leaves, Asn synthetase (ASN) isoforms are reciprocally regulated by light. Interestingly, the isoform that is highly expressed during seed maturation, ASN1, is induced in the dark indicating a possible regulation of seed Asn synthesis by light availability (Lam et al., 1998, eFP Browser, Winter et al., 2007). It has been postulated that in soybean seeds Asn has a role in storage product accumulation and might act as signal of the internal nitrogen status (Hernández-Sebastià et al., 2005). Gln synthetase (GLN) is regulated at the transcriptional as well as post-translational level in response to nitrogen availability and environmental stress (Bernard and Habash, 2009). We detected one of the dark inducible isoforms, GLN1-1, exclusively in the dark grown seeds indicating that seed Gln synthesis might be up-regulated by darkness. However, since the abundance of the light inducible isoform GLN1-2 was decreased it is not possible to draw a final conclusion about the resulting rate of Gln synthesis in dark grown compared to control seeds. An additional likely explanation for the rise in Asn and Gln levels might be the lowered demand for storage protein biosynthesis in the dark.

Our results reveal clear differences between the effect of extended darkness on amino acid metabolism in leaves and seeds. In vegetative tissues, proteins are degraded in situations of carbohydrate starvation such as darkness to produce amino acids as alternative substrates for mitochondrial respiration (Araujo et al., 2011). The branched-chain amino acids Val, Leu, and Ile as well as Lys are particularly relevant in this functional context since their catabolic pathways produce high amounts of ATP and directly transfer electrons into the respiratory chain. We did not find any indication for either an accumulation of these low abundant amino acids or a specific induction of their degradation pathways, which are both characteristic features of the carbohydrate starvation response in leaves. The metabolite profiles indeed revealed only a mild shortage of carbohydrates in developing seeds in the absence of photosynthesis, which was expected since sugars are delivered from the mother plant (Melkus et al., 2009). In contrast to leaves, which use protein degradation as a short-term survival strategy in the absence of photosynthesis and would eventually die in the dark, Arabidopsis seeds are able to complete their development without light and remain viable. Many plant species such as sunflower, maize, and castor bean produce non-green, entirely heterotrophic seeds (Borisjuk and Rolletschek, 2009).

### Physiological Function of ETHE1 in Seeds

#### ETHE1 Affects Seed Amino Acid Homeostasis

The functional context of ETHE1 in leaves, the use of amino acids as alternative respiratory substrates, is according to our results most likely not relevant during embryo development. In agreement with this result the amino acid profile of *ethe1-1* seeds did not show the characteristic changes present in the leaves, such as an accumulation of branched-chain amino acids and Lys (Krüßel et al., 2014; Höfler et al., 2016). We detected a rather general increase of free amino acids in *ethe1-1* seeds compared to the wild type, which has also been described for seeds of mutant lines with different defects in branched-chain amino acid degradation (Gu et al., 2010; Peng et al., 2015). In particular, Ala and Gly were strongly increased (about fivefold) in *ethe1-1* seeds during early development (5 DAP) independent of the light regime. At this stage, the endosperm still represents a major part of the seed so that the differences detected might be present in in the endosperm, the embryo, or both amino acid pools. The endosperm is the first sink within the seed, and Ala is one of the amino acids imported from the mother plant (Baud et al., 2008). The endosperm amino acid concentration is high during early developmental stages and decreases during seed filling (Melkus et al., 2009). However, the reason for the specific accumulation of Ala and Gly in young *ethe1-1* seeds is presently unknown.

#### ETHE1 Is Required for Cell Organization and Cell Wall Establishment

Knock-out of the sulfur dioxygenase ETHE1 in Arabidopsis leads to an arrest in embryo development at the heart stage. The earliest effects observed were fewer endosperm nuclei as well as defective cellularization and premature degeneration of the endosperm (Holdorf et al., 2012). Several lines of evidence indicate that defects in endosperm cellularization also contribute to the delay in embryo development in the ETHE1 knock-down line *ethe1-1* used for this study. Since these defects are not lethal, they can be studied using homozygous plants in order to unravel the mechanistic background. Phenotype analysis revealed that the endosperm of developing *ethe1-1* seeds is composed of enlarged irregularly shaped cells. We found increased amounts of actin and tubulin indicating that the initial step of endosperm cellularization, development of a radial microtubule system to organize the endosperm into distinct nuclear cytoplasmic domains and provide even spacing between the nuclei, was still present and maybe even induced in the mutant seeds. The next steps would be migration of golgi-derived vesicles along these microtubules, establishment of a tubular network across the division plane that further develops into a coherent cell plate, and finally maturation of the new plasma membrane and cell wall (Berger et al., 2007; Otegui, 2007). The consistent down-regulation of proteins associated with vesicle transport together with the strong effect on cell wall metabolic proteins in *ethe1-1* seeds shows that the defect in persulfide oxidation most likely interferes with one or several of these processes. It is tempting to speculate that the accumulation of free hydroxyproline in *ethe1-1* seeds might be due to an increased turnover of glycoproteins from incompletely assembled or misarranged cell walls. A high turnover of cell wall elements and defective cell structure establishment might not only affect endosperm cellularization but also nutrient transport to the embryo during development of *ethe1-1* seeds. This secondary effect has been postulated for the *endosperm-defective 1* mutant, which is deficient in a microtubule-associated protein essential for endosperm cellularization (Pignocchi et al., 2009). Insufficient nutrient supply might in turn lead to the formation of giant mitochondria in *ethe1-1* as well as in ETHE1 knock-out seeds (Holdorf et al., 2012). Recently it has been shown that in leaves the establishment of giant mitochondria is induced by darkness, at reduced carbon levels and under hypoxia (Jaipargas et al., 2015).

#### Possible Role of ETHE1 in ABA Signaling

Abscisic acid has critical functions during several steps in seed development, germination, and early seedling growth. ABA levels in the siliques peak during early seed maturation and are maximal at 9 DAP (Kanno et al., 2010). Our proteome data show that at this developmental stage ETHE1 deficiency leads to a specific decrease in the abundance of ABA inducible proteins indicating that the sulfur dioxygenase might have a presently unknown function in ABA signal transduction. Another indication for a functional connection is the strong induction of ETHE1 expression by ABA (Supplementary Figure S3, eFP Browser, Winter et al., 2007). In wild type Arabidopsis plants ABA inhibits germination and also leads to an arrest of seedling growth at a checkpoint immediately after the emergence of the radicle from the seed coat probably to keep the germinated seed in a quiescent state if environmental conditions are unfavorable for the survival of the young seedling (Lopez-Molina et al., 2001). In fact, at the low concentration used for this study (1 μM), ABA is rather an inhibitor of early seedling growth than of germination. ABA signaling interacts closely with the antagonistic phytohormone gibberellin (GA) in a complex network involving diverse types of regulators (Shu et al., 2018a,b). The transcription factor ABI4 enhances ABA signal transduction and represses GA biosynthesis thus acting as a key player for precisely adjusting ABA and GA responses (Shu et al., 2018b). ABI4 deficient mutants as well as overexpression lines for cytokinin-inducible response regulators show a decreased ABA sensitivity during early seedling growth comparable to the results we obtained with *ethe1-1* (Huang et al., 2017). Thus, ETHE1 might act as either a positive regulator of ABA signaling or a negative regulator of gibberellin and cytokinin signaling. Interestingly, the transcription factor HY5 responsible for the activation of many light regulated genes is also a transcriptional activator of the ABA-responsive transcription factor ABI5 so that light positively regulates the ABA response (Chen et al., 2008). Thus, the stronger effect of shading on the development and the proteome of *ethe1-1* compared to wild type seeds we detected in this study might be connected to defects in ABA signaling. Mutations in ABA2, an enzyme involved in the biosynthesis of ABA, lead to a delay in endosperm cellularization and an increased embryo cell number (Cheng et al., 2014). In barley, ploidy levels of the developing endosperm are inversely correlated to the ABA content suggesting an influence of ABA on cell-cycle regulation (Sreenivasulu et al., 2010). Thus, the defects in endosperm cellularization detected in the *ethe1-1* seeds might also be a consequence of the postulated disturbance in the ABA signaling process.

The primary reason for the different effects detected in *ethe1-1* seeds could be an accumulation of persulfides, which are the substrate of the sulfur dioxygenase ETHE1. Deficiency of the cysteine desulfhydrase DES1 has been shown to interfere with several metabolic pathways due to decreased production of the signaling molecule hydrogen sulfide (Romero et al., 2013; Gotor et al., 2015). Interestingly, one of the effects in the *des1* mutant is a block in ABA induced stomatal closure indicating a function of sulfide in ABA signal transduction (García-Mata and Lamattina, 2010). Hydrogen sulfide and also persulfide signaling might be mediated by persulfidation of specific cysteine residues in proteins. More than 2000 Arabidopsis proteins have been shown to be persulfidated under control conditions, and among them were ABA receptors as well as cytoskeleton proteins (Aroca et al., 2017). Further research will be required to elucidate the potential role of the mitochondrial sulfur dioxygenase ETHE1 in sulfur signaling and protein persulfidation.

## Data Availability

All datasets generated and analyzed for this study are included in the manuscript and the Supplementary Files.

## Author Contributions

CL and TH designed the research. CL produced the plant material, performed phenotypic analysis including light microscopy and performed shotgun proteomics. LB contributed electron microscopy. HR and NH analyzed the composition of mature seeds. SB tested germination rates in the presence of ABA. TT and AF performed metabolite profiling. H-PB contributed to the scientific context. CL and TH wrote the manuscript. SB, LB, HR, AF, and H-PB contributed to manuscript revision.

## Conflict of Interest Statement

The authors declare that the research was conducted in the absence of any commercial or financial relationships that could be construed as a potential conflict of interest.
