# Supplementary material for: The Role of Persulfide Metabolism During Arabidopsis Seed Development Under Light and Dark Conditions
Source: Front Plant Sci. 2018 Sep 19;9:1381. doi: 10.3389/fpls.2018.01381 (PMC6156424; doi:10.3389/fpls.2018.01381)
Supplement: Supplementary file 1 [file Data_Sheet_1.PDF]

Supplementary Figures

The role of persulfide metabolism during Arabidopsis seed development under light and dark conditions

Christin Lorenz, Saskia Brandt, Ljudmilla Borisjuk, Hardy Rolletschek, Nicolas Heinzel, Takayuki Tohge, Alisdair R. Fernie, Hans-Peter Braun, Tatjana M. Hildebrandt*

*** Correspondence:** Dr. Tatjana Hildebrandt, hildebrandt@genetik.uni-hannover.de

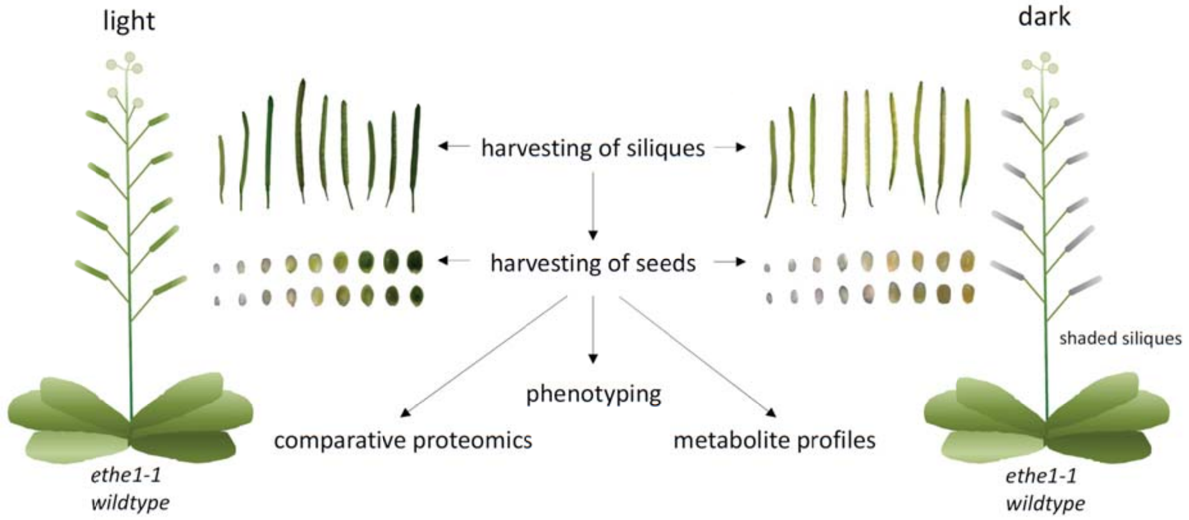

Supplementary Figure S1: Experimental design. *A. thaliana* wild type (ecotype Columbia) and *ethe1-1* plants were grown in a climate chamber under long-day conditions (16h light/8h dark, 22°C, 85 µmol s^{-1} m^{-2} light intensity and 65% humidity). Flowers were labeled at the day of pollination. Subsequently siliques were harvested from 1 to 9 DAP. For dark treatment, siliques were shaded with aluminium foil 24 hours after flower tagging while the rest of the plant and control siliques were grown under normal light conditions. Seeds were investigated by phenotyping, comparative proteomics and measuring metabolite profiles.

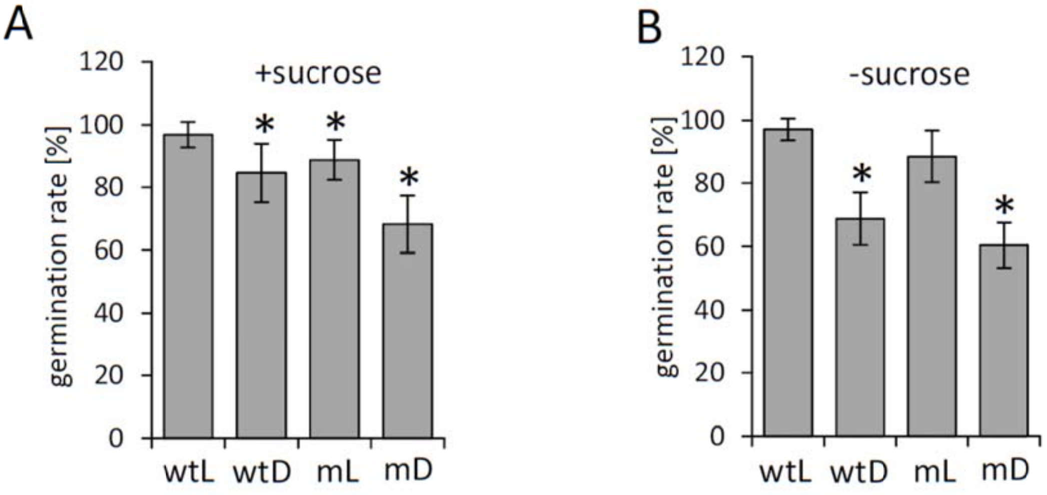

Supplementary Figure S2: Germination rates. Sterilized seeds of wild type and *ethe1-1* grown under light and dark conditions were sown per plate (3 replicates per sample) on MS-medium [60 mM sucrose, 1% Agar, 0.5% MS-medium (Duchefa), pH 5.7-5.8 with KOH] and MS medium without sucrose and incubated for another 2 days at 4°C in the dark. Afterwards the plates were placed to a growth chamber (24°C, 16 h light/8 h dark). After 72h germinated seeds were counted. A seed is considered to be germinated when the radicle ruptures the endosperm and the testa.

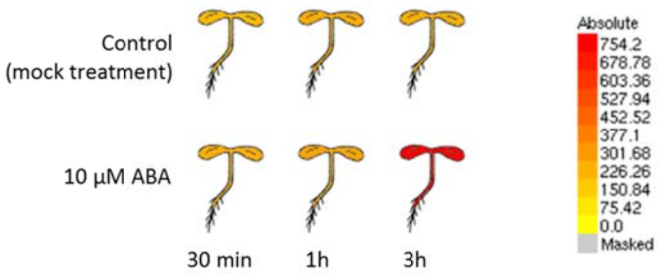

Supplementary Figure S3: Induction of ETHE1 expression by abscisic acid (ABA). Expression levels of AT1G53580 in 7 day old wild type Arabidopsis seedlings grown in liquid MS medium under continuous light conditions at 23°C after treatment with 10 µM ABA. The figure was generated using the Arabidopsis eFP Browser (http://bar.utoronto.ca/efp/cgi-bin/efpWeb.cgi, Winter et al. 2007), the microarray datasets are available at TAIR (https://www.arabidopsis.org/): ExpressionSet:1007964750 "ABA time course in wild type seedlings".

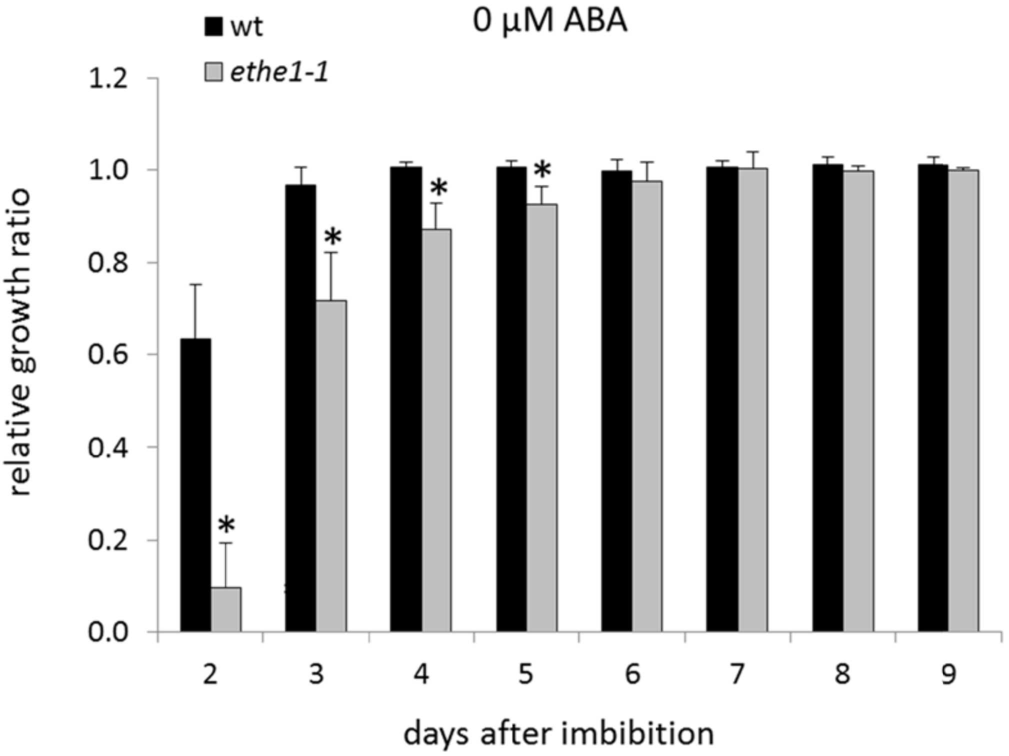

Supplementary Figure S4: Relative growth ratio of wild type (wt) compared to *ethe1-1* seedlings on control agar plates.

The growth ratio of seedlings on agar plates containing 0 µM ABA was calculated as the percentage of germinated plants that had also developed leaves. All data points were normalized to the growth ratio of *ethe1-1* seedlings at 9 days after imbibition. Asterisks indicate significant differences to the wild type based on a Student´s t-test (p-value ≤0.05).
